# Are sleeping site ecology and season linked to intestinal helminth prevalence and diversity in two sympatric, nocturnal and arboreal primate hosts (*Lepilemur edwardsi* and *Avahi occidentalis*)?

**DOI:** 10.1186/s12898-018-0178-8

**Published:** 2018-07-13

**Authors:** May Hokan, Elke Zimmermann, Ute Radespiel, Bertrand Andriatsitohaina, Solofonirina Rasoloharijaona, Christina Strube

**Affiliations:** 1 Institute for Parasitology, University of Veterinary Medicine Hannover, Buenteweg 17, 30559 Hannover, Germany; 2 Institute of Zoology, University of Veterinary Medicine Hannover, Buenteweg 17, 30559 Hannover, Germany; 3 Département de Biologie Animale et Écologie, Faculté des Science, Université de Mahajanga, 401 Mahajanga, Madagascar

**Keywords:** Helminths, Milne-Edward’s sportive lemur, Western woolly lemur, Strongylida, Oxyurida, Arboreality, Seasonality, Sleeping site ecology

## Abstract

**Background:**

Various factors, such as climate, body size and sociality are often linked to parasitism. This constrains the identification of other determinants driving parasite infections. Here, we investigate for the first time intestinal parasites in two sympatric arboreal primate species, which share similar activity patterns, feeding ecology, body size and sociality, and cope with the same climate conditions, but differ in sleeping site ecology: the Milne-Edward’s sportive lemur (*Lepilemur edwardsi*) and the Western woolly lemur (*Avahi occidentalis*). Comparison of these two species aimed to test whether differences in sleeping sites are related to differences in parasite infection patterns. Additionally, gender and seasonal factors were taken into account. Animals were radio-collared to record their sleeping site dynamics and to collect fecal samples to assess intestinal parasitism during both the dry and the rainy season.

**Results:**

Only low parasite diversity was detected, which is attributable to the strict arboreal lifestyle of these lemurs, limiting their contact with infective parasite stages. *L. edwardsi*, which sleeps in tree holes and repeatedly uses the same sleeping site, excreted eggs of strongyle and oxyurid nematodes, whereby strongyles always occurred in coinfection with oxyurids. In contrast, *A. occidentalis*, which sleeps on open branches and frequently changes sleeping sites, only excreted eggs of strongyle nematodes. This difference can be attributed to a potential favorable environment presented by tree holes for infective stages, facilitating parasitic transmission. Additionally, Strongylida in *A. occidentalis* were only observed in the rainy season, suggesting an arrested development during the dry season in the nematodes’ life cycle. Males and females of both lemur species showed the same frequency of parasitism. No differences in body mass of infected and non-infected individuals were observed, indicating that the animals’ body condition remains unaffected by the detected gastrointestinal parasites.

**Conclusions:**

The comparison of two primate hosts with a very similar lifestyle suggests an influence of the sleeping site ecology on intestinal parasites. In *A. occidentalis* there was a clear seasonal difference in strongyle egg excretion. These results improve our understanding of the parasite ecology in these endangered primate species, which may be critical in the light of species conservation.

**Electronic supplementary material:**

The online version of this article (10.1186/s12898-018-0178-8) contains supplementary material, which is available to authorized users.

## Background

Parasites can impact animal populations by reducing their host’s condition [1], susceptibility to predation [2] or reproductive potential [3] and therefore fitness. Although intestinal parasites often act as commensals, they may become pathogenic in immunocompromised hosts [4]. Furthermore, when the animal is subject to chronic stress, for example through anthropogenic impact or temperature variations, e.g. related to climate change [5], parasite virulence can be increased [6, 7]. Parasites may also act as stressors themselves and facilitate coinfections with other pathogens [8]. Therefore, studies on patterns of parasitism in wild populations are needed, especially in host species that are increasingly endangered by anthropogenic threats.

Patterns of parasitic infections may be influenced by the host’s ecology [9]. Along with diet, group size, density, ranging behavior and grooming, sleeping site choice may influence parasite load [10–12]. A previous study on primates by Hausfater and Meade [13] has postulated that sleeping site ecology may have a direct influence on parasite infection: yellow baboons (*Papio cynocephalus*) appear to avoid potential infection through their own fecal emission by regularly rotating their sleeping sites. However, the results of a more recent study on baboons in the same area found intervals of vacancy of the sites to be too short to reduce the risk of sharing parasites [14]. The selection of a sleeping site in Capuchin Monkeys (*Cebus apella nigritus*) is mainly influenced by the degree of safety it provides by reducing accessibility for predators [15]. Tamarins (*Leontopithecus rosalia*), for example, use tree cavities, which are big enough to permit their entry but not that of predators [16].

Sleeping sites, such as tree holes, can also provide shelter from unfavorable weather conditions so that inhabitants spend less energy on thermoregulation [17]. Another benefit of sleeping in tree holes is the decreased exposure to vectors leading to reduced rates of mosquito bites and thus to a lower infection risk of transmitted diseases such as malaria [18].

Using tree holes as sleeping sites also bears some disadvantages: the usage of shelters by multiple individuals can lead to increased parasite transfer [19, 20]. Also, tree holes are limited compared to open sleeping sites, leading to a competition for such sites and to a higher rate of reutilization, which in turn leads to higher parasite infection rates [21, 22]. Finally, a higher site fidelity, which can result from a preference for rare high quality sites [14] or from smaller home range sizes [23], may to lead to increased infection rates because of nest contamination. Another ecological trait worth mentioning in relation with parasitic infections is arboreality. In arboreal species, water- and soil-borne parasitic infection rates were found to be lower than in terrestrial species [24]. Purple-faced langurs (*Semnopithecus vetulus*) for instance avoid contaminated soil and water due to their arboreal habits, protecting them from acquiring *Cryptosporidium* sp. [25] and several arboreal mammal species in French Guinea show significantly lower prevalences of *Toxoplasma gondii* [26].

Environmental factors, such as temperature and rainfall, may also influence parasite occurrence. For example, humidity favors survival and hatching of helminth eggs, which may lead to an increase of parasite load in the rainy season [27, 28]. Nevertheless, in some cases a lower parasite richness is observed in rainy season due to heavy rainfalls which can have “wash-out” effects on free living stages of nematodes decreasing fecal contamination [29].

Furthermore, sex differences in intestinal parasite infections have been documented, as the prevalence and infection intensity is occasionally reported to be higher in males than females [30–32]. This could be due to the immunosuppressive effect of testosterone and/or to higher exposure to parasites due to sex-specific behavior, such as aggression, foraging and grouping [33, 34]. However, prevalence of some parasite species may also be higher in females [35] and other studies showed no sex differences [36]. Hence, it is important to explore the possible influence of host sex and season in studies on parasite load in wildlife.

Other factors linked to parasitic infections in wild hosts are body size, group size, population density, sociality, feeding ecology and habitat [9–12]. Studies on the influence of sleeping sites on parasite infections in primates are so far limited [13, 18, 37, 38] and challenging due to the interaction of multiple factors. Thus, to assess the influence of a single factor, such as sleeping site ecology, the other elements need to be controlled. This can be achieved by studying two host species sharing similar influential factors like activity patterns, feeding ecology, body size, sociality and habitat. Here we investigated infection patterns with intestinal parasites in a wild population of two endangered Malagasy primates, the Milne-Edward’s sportive lemur (*Lepilemur edwardsi*) and the Western woolly lemur *(Avahi occidentalis*). These nocturnal lemurs live in northwestern Madagascar with a home range of 1 ha and an estimated density of 60 individuals/km^2^ for *L. edwardsi* [39, 40] and a home range of 1.96 ha and a density of 67 individuals/km^2^ for *A. occidentalis* [40–42]. Both species are pair-living, folivorous and have a similar body size of approximately 900 g [43–45] but differ in their sleeping site ecology. Sportive lemurs sleep in tree holes and individuals show high sleeping-site fidelity, whereas woolly lemurs sleep on open branches and shift their sleeping sites frequently [46, 47]. Thus, we aimed to examine the influence of sleeping site ecology, seasonality and sex on intestinal parasite infection patterns in two lemur hosts with the same habitat, feeding ecology, density, activity, sociality and body size. We hypothesize that *L. edwardsi* shows a higher prevalence because they frequently revisit the same tree holes, facilitating transmission of infective parasite stages. We also expect a general influence of season with a higher prevalence during the rainy season. Additionally, if sex affects the infection status, we expect males of both species to excrete more eggs than females. Finally, we take into account the body mass of the hosts as an indicator for the hosts’ body condition, to assess whether the presence of endoparasites compromises animals’ health [1, 4, 48].

## Methods

### Study site

**Fig. 1:**
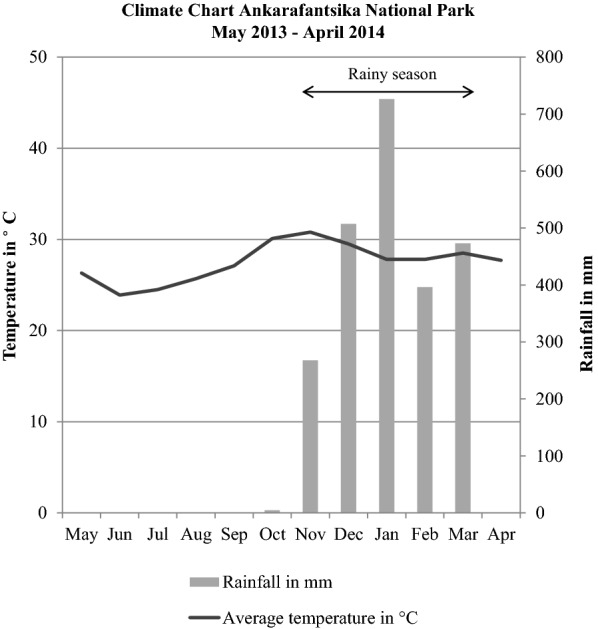
Temperature and rainfall in the Ankarafantsika National Park during the sampling year

The study was conducted in the Jardin Botanique A (JBA), a 30.6 ha forest parcel located at 16° 19′ S, 46° 48′ E in the Ankarafantsika National Park in northwestern Madagascar. The park contains dry deciduous forest and is subject to pronounced seasonality, with a dry season from May to October and a hot, rainy season from November to April (Fig. 1).

### Animal hosts

Lemur capturing included a total of 26 individuals of *L. edwardsi*, 15 of which were recaptured at least once during the study, and 22 individuals of *A. occidentalis,* seven of which were recaptured. *L. edwardsi* was caught directly in tree holes before immobilization with a combination of ketamine (Ketanest^®^ Pfizer Deutschland GmbH, Berlin, Germany, 25 mg/ml) and xylazine (Rompun^®^, Bayer AG, Leverkusen, Germany, 20 mg/ml) for collaring. *A. occidentalis* were remotely immobilized with the same drug mixture by using a blowpipe and 1 ml cold air pressure darts (Telinject^®^). Dosages based on estimated body weights were 10 mg/kg ketamine and 0.5 mg/kg xylazine. During immobilization, a total of 13 individuals of *L. edwardsi* were radio-collared and nine individuals of *A. occidentalis* were equipped with a radio-transmitter backpack to avoid impairment of their natural marking behavior with throat glands [49]. All radio-transmitters were TW-3 tags (Biotrack, UK). The tagged individuals were chosen randomly. Additionally, all animals were microchipped (ID100 Microtransponder, Trovan^®^), sexed and weighed (5 kg-balance, AEG). All procedures were approved by the Ministère de l’Environnement, de l’Ecologie et des Forêts and Madagascar National Parks (MNP) and necessary research permits were obtained from the competent Malagasy authorities (License N° 167/13/MEF/SG/DGF/DCB.SAP/SCB obtained on the 13th of July 2013 and N°072/14 obtained on the 12th of March 2014).

### Sleeping sites

Sleeping sites were determined during two periods, from July to October 2013 representing the dry season as well as from March to May 2014 representing the rainy season. Radio-collared animals were located daily in the morning and their daytime sleeping site was marked with a numbered flag. Additionally, the GPS coordinates of the tree and the sleeping site type (tree hole or branch) were noted. The number of daytime sleeping sites used was determined for each individual (Additional file 1) and the mean number of days spent in one sleeping site was calculated. The latter data was used to compare the frequency of sleeping site rotation of *L. edwardsi* and *A. occidentalis* using a Mann–Whitney U Test (IBM SPSS Statistics, version 23).

### Sample collection

Throughout two sampling periods (July–October 2013 and March–May 2014), 86 fecal samples from *L. edwardsi* (43 from the dry and 43 from the rainy season) and 74 samples from *A. occidentalis* (33 from the dry and 41 from the rainy season) were collected (Table 1, Additional files 2 and 3). During animal anesthesia, samples could be collected directly from the animal. However, from most specimens fecal samples were gained non-invasively by collection from the ground during nocturnal focal animal observations. A total of 111 samples were obtained from tagged and 49 samples from untagged animals. The firm, small droppings were collected as a whole, labeled individually and stored in 90% ethanol. In total, samples from 28 individual *L. edwardsi* and 29 individual *A. occidentalis* were obtained, which approximately represent the whole population present in JBA according to the densities reported by Ganzhorn [41] and Warren and Crompton [40] matching personal observations.

**Table 1:** Sample size and the number of sampled individuals in square brackets per season and per species

Host species	Dry season	Rainy season
*L. edwardsi*	43 [23]	43 [19]
*A. occidentalis*	33 [18]	41 [16]

### Fecal flotation and identification of parasitic stages

All fecal samples were processed by the flotation method using saturated sodium chloride (NaCl, specific gravity 1.2) as flotation solution. In brief, feces were weighed, grind in a mortar and transferred to a 15 ml centrifuge tube. Two-thirds of the tube were filled with distilled water. The fecal content was homogenized by shaking and centrifuged at 800*g* for 10 min. After centrifugation, the supernatant was removed and pelleted feces were poured through a tea strainer (mesh size 0.5 mm) into a 50 ml tube. Feces remaining in the tea strainer were washed with saturated NaCl until the 50 ml tube was full. A microscope cover slip covering the entire opening was placed on top of the tube and transferred to a microscope slide after 30 min. For each sample, the slide was scanned and parasite eggs were counted using the 10× objective. The 40× objective was used to identify parasites based on morphological parameters using descriptions from Irwin and Raharison [50]. Parasites were photographed with an Olympus CAMEDIA C-5050 Zoom digital camera, then visualized and measured with the cell^B Image Acquisition Software (version 3.1; Olympus Soft Imaging Solutions).

### Statistical analyses of parasites

First, the overall prevalence of each parasite type for *L. edwardsi* and *A. occidentalis* was analyzed. For this purpose, each individual entered the calculation only once per season and an animal was considered positive, if at least one of its samples from that season was positive. For subsequent analyses, the information content of multiple samples per individual and season was included. To assess the influence of host species, sex and season on the probability of being infected with a certain egg type, a generalized linear mixed model (GLMM) with binomial error structure and logit link function was implemented. The models contained the variables “species” (*L*. *edwardsi*, *A. occidentalis*), “sex” (male, female) and “season” (dry, rainy) as fixed factors. Animal ID was included as a random factor to account for the fact that many individuals (56.1%) contributed more than one sample. Full models were tested containing all factors, followed by evaluation of the significance of each factor. The exponential function was used to calculate the extent of difference between two groups when a factor was significant. Except when examining the factor host species, all analyses were conducted separately for *L*. *edwardsi* and *A. occidentalis* and for each parasite morphotype, in order to infer whether the observed effect is only present in one or in both host species. In *L. edwardsi*, the number of tree holes used by each radio-collared individual was tested additionally. In *A. occidentalis*, the factor season was not evaluated statistically because no intestinal helminths were ever found in the dry season. The analyses were performed in R v.3.2.2 [51] using the package lme4 [52].

Fecal egg counts (FEC) as a measurement of infection intensity were not analyzed further, since the observed oxyurid parasites do not excrete their eggs in the intestinal lumen, but deposit them on the perianal region, while egg excretion of strongyle nematodes is not necessarily correlated with parasite intensity.

As an indicator for the host’s condition, we examined the host’s body mass [48]. Using a Mann–Whitney U Test (IBM SPSS Statistics, version 23), it was tested whether there was a difference in body mass between individuals excreting helminth eggs and those who did not. Only body mass data from adult individuals and from the rainy season was taken into account, as during this season no pregnant females are present, and there is no restriction in food supply.

## Results

### Sleeping sites

Sleeping sites of 13 individuals of *L. edwardsi* and 9 individuals of *A. occidentalis* were determined over a total of 130 days. Due to losses caused by predation or animals that could not be located on some days, sleeping sites were determined at an average of 98 days (min: 36, max: 129 days) for *L. edwardsi* and 68 days (min: 24, max: 119 days) for *A. occidentalis* (Additional file 1).

Sleeping sites of *L. edwardsi* (N = 13) were tree holes in 100% of the observations and an individual sleeping site was used for a mean of 25 days (range 1–107 days, Additional files 4 and 5). They slept solitarily, in pairs or in a family group. *A. occidentalis* (N = 9) used the same sleeping site for a mean of 4 days (range 1–28 days, Additional files 6 and 7). Woolly lemurs always slept in pairs or families huddled together on branches or tree forks. The individual average usage frequency of a sleeping site was significantly different between the two lemur species (Mann–Whitney *U* = 0, *n*_1_ = 13, *n*_2_ = 9, *p* = 0.0002).

### Parasite diversity and prevalence

**Fig. 2:**
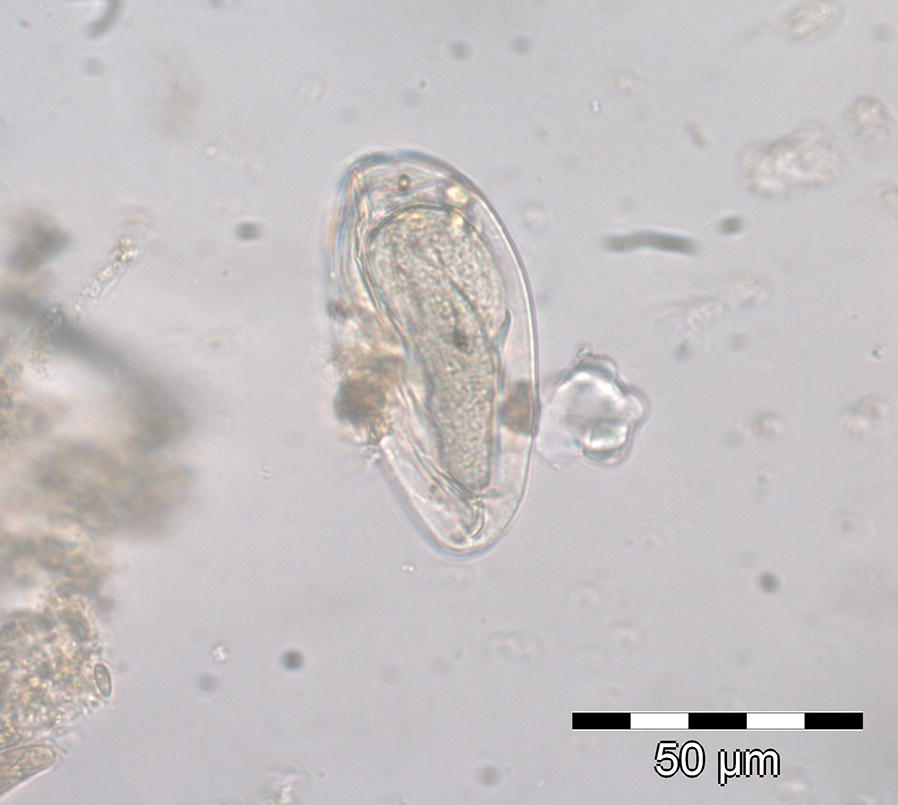
Oxyurid egg (*Lemuricola* spp.) of *L. edwardsi*

**Fig. 3:**
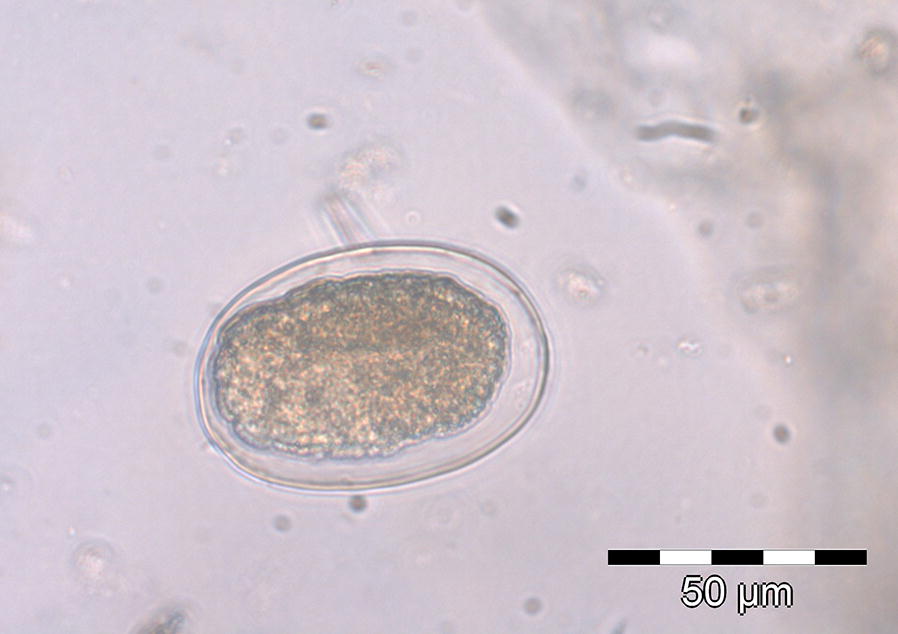
Strongyle egg of *L. edwardsi*

Both lemur species excreted helminth eggs, but no protozoal stages were detected. Fecal samples of *L. edwardsi* contained oxyurid (*Lemuricola* spp.) and strongyle eggs (Figs. 2 and 3). Strongyle eggs were only recovered from individuals, which were also infected with *Lemuricola* spp. (in six samples), whereas 30 samples (34.9%) were positive for *Lemuricola* spp. only. The asymmetrical, thin-walled *Lemuricola* eggs were all similar in shape and size, with a mean length of 71.4 µm (SD 5 µm) and a mean width of 32.3 µm (SD 4 µm) (N = 50) and contained an embryo (Fig. 2). Strongyle eggs from *L. edwardsi* measured 71.1 µm (SD 5 µm) by 43.8 µm (SD 4 µm) in width (N = 6) and contained a morula or larva (Fig. 3). Fecal samples of *A. occidentalis* contained only strongyle eggs, resembling those from *L. edwardsi* and measuring 69.9 µm (SD 4 µm) in length by 43.0 µm (SD 3 µm) in width (N = 40).

The overall prevalence in *L. edwardsi* was 64.3% (N = 28 individuals) for *Lemuricola* spp. and 21.4% (N = 28 individuals) for Strongylida. Eggs were shed in both seasons (Table 2). For *A. occidentalis,* the overall Strongylida prevalence was 20.7% (N = 29 individuals), but egg shedding was only observed during the rainy season (March–May 2014).

**Table 2:** Number of infected individuals out of the number of sampled *L. edwardsi* or *A. occidental* in each season with seasonal prevalences in brackets

Host species	Dry season		Rainy season	
	*Lemuricola* spp.	Strongyles	*Lemuricola* spp.	Strongyles
*L. edwardsi*	11/23 (47.8%)	2/23 (8.7%)	11/19 (57.9%)	4/19 (21.1%)
*A. occidentalis*	0/18 (0.0%)	0/18 (0.0%)	0/16 (0.0%)	6/16 (37.5%)

### Effect of host species, sex and season on parasite infection

A statistically significant effect of host species on parasite infection status was observed. According to the exponential function, the probability of shedding parasite eggs was three times higher for *L. edwardsi* than for *A. occidentalis* (CI 1.3–8.6, Table 3). In addition, season clearly had an influence on strongyle infections in *A. occidentalis*, showing 0.0% prevalence in the dry season, but 37.5% prevalence in the rainy season (Table 2). However, this was not the case for *L. edwardsi*, as likelihood of infection was not significantly affected by season for neither parasite morphotype (Table 3). The number of tree holes used as sleeping sites by *L. edwardsi* did not significantly affect their likelihood of infection with either parasite. Finally, neither host species showed a significant effect of sex on parasite infection; 37.2% of males and 44.2% females of *L. edwardsi* as well as 75.0% of males and 69.2% females of *A. occidentalis* were tested positive.

**Table 3:** Results of GLMMs testing the influence of animal species, sex, season and number of used tree holes on helminth prevalence

Measure	Term	Estimate	Standard error	z	*p* value
Overall prevalence	Intercept	− 1.47	0.39	− 3.78	< 0.001*
	Species	1.08	0.45	2.43	0.015*
*Lemuricola* spp. prevalence in *L. edwardsi*	Intercept	0.98	0.72	1.35	0.178
	Sex	− 0.44	0.51	− 0.85	0.395
	Season	− 0.30	0.57	− 0.57	0.572
	Tree holes	− 0.45	0.29	− 0.15	0.125
Strongylida prevalence in *L. edwardsi*	Intercept	− 2.68	1.15	− 2.34	0.019*
	Sex	− 1.94	1.20	− 1.62	0.106
	Season	− 0.57	1.95	− 0.60	0.106
	Tree holes	0.57	0.47	1.20	0.229
Strongylida prevalence in *A. occidentalis*	Intercept	− 4.83	4.34	− 1.11	0.266
	Sex	0.87	1.90	0.46	0.649

* Significant *p* values (≤ 0.05)

### Parasites and body mass

In both host species body mass of individuals shedding eggs was not significantly different from those who did not excrete eggs (*L. edwardsi*: Mann–Whitney *U *= 28.5, n_1_ = 10, n_2_ = 6 *p* = 0.88; *A. occidentalis* Mann–Whitney *U *= 9, n_1_ = 8, n_2_ = 5, *p* = 0.13) (Additional file 8).

## Discussion

Parasite transmission modes and the influence of ecological factors on infection patterns are still unclear in many ecosystems. In this study, intestinal parasite infection patterns in *L. edwardsi* and *A. occidentalis*, which occur in sympatry in Ankarafantsika National Park in north-western Madagascar, were examined to compare their association with host traits such as sleeping site ecology and sex, as well as with environmental factors (season). In general, both species harbored a low diversity of intestinal helminths and no protozoal stages were detected. *Lepilemur edwardsi* excreted strongyle as well as oxyurid eggs, a result comparable to the findings of previous studies on *Lepilemur* spp. [53–56]. By contrast, *A. occidentalis* excreted exclusively strongyle eggs, which is also consistent with findings of previous studies [57, 58]. Although the strongyle eggs found in *A. occidentalis* resemble those found in *L. edwardsi*, it is quite possible that they belong to different parasite species. Molecular analysis would be needed to clarify this matter. Neither host species showed a difference in body mass between individuals excreting these helminth eggs and those who did not. If not resulting from low statistical power due to the small sample size, this might be an indication that the animals’ health is not compromised by the presence of the observed strongyle and oxyurid nematodes. Furthermore, the intensity of infection may be too low to exert any adverse effects. Unfortunately, determination of reliable infection intensity was not possible in our study, since egg excretion of strongyle nematodes is not necessarily correlated with infection intensity and egg excretion of oxyurid nematodes occurs in the perianal region rather than the intestinal lumen. Thus, the determined percentage of *Lemuricola* spp. infections might not reflect the true but rather an underestimated prevalence.

Beside strongyles, only one other type of intestinal helminth, the tapeworm *Bertiella lemuriformis*, was previously noted in *Avahi* spp. [59]. This noticeably low parasite species richness may have several ecological reasons as discussed below, but might also be a consequence of the very limited number of studies existing on parasites in *Lepilemur* and *Avahi* species. Even though the present study may be hampered by the moderate sample numbers of 28 *L. edwardsi* and 29 *A. occidentalis* specimens, these individuals nearly represent the whole *Lepilemur* and *Avahi* population of the 30.6 ha study site [40, 41], as both populations have suffered a strong decline due to poaching during the last years. Therefore, if other parasite infections are present in this population, they should be visible in this almost complete population sample.

One ecological mechanism leading to reduced parasite species richness may be the strict arboreal lifestyle of these primates, who leap from tree to tree avoiding ground contact [60]. As many parasites develop their infective stage in the environment, transmission is reduced when contact with the contaminated forest floor is limited. This has also been observed in Verreaux’s sifaka (*Propithecus verreauxi*), another arboreal lemur species [61, 62]. Remarkably, it has been demonstrated that threatened host taxa harbor fewer parasite species because of the host’s lower population size and range area [63]. As both of the studied lemur populations probably underwent population bottlenecks before this study, the low parasite richness may also result from the direct loss of parasite species, meaning that parasites can “fade out” and they can go extinct long before their host [64]. The folivorous diet of *L. edwardsi* and *A. occidentalis*, may also explain the low parasite species richness found in this study as it prevents infection from parasite genera using invertebrates as intermediate hosts. Another possible explanation for the low parasite species richness may be the relatively small group size as parasite transmission may increase with the number of animals in a group [12]. Furthermore, a monogamous lifestyle limits the number of sexual and grooming partners, reducing chances of contamination with parasites that are amongst others transmitted through direct contact, such as *Lemuricola* spp. Both aspects, small groups as well as monogamy apply to *A. occidentalis* and *L. edwardsi*, which were mainly observed sleeping in pairs in the present study, restricting close contact to multiple individuals.

One of the main objectives of this study was to test the influence of sleeping site ecology on endoparasite prevalence and species richness. As expected, *L. edwardsi*, which sleeps in tree holes, showed higher prevalence and parasite species richness than *A. occidentalis*, which sleeps on open branches. The population density and social organization of these primate species is similar, both species live in pairs or small groups and are monogamous, they share their habitat with identical climate conditions, have the same activity pattern and a similar feeding ecology, so that all these factors cannot explain the differences observed between the two species. The comparison of these hosts indicates that the specific sleeping site ecology of *L. edwardsi* may promote intestinal parasitism. The used tree holes are often frequented by two or three *L. edwardsi* individuals at a time, resulting in close physical contact between individuals. Oxyurid nematodes, a parasite group that was only found in *L. edwardsi* and not in *A. occidentalis*, attach their eggs to the host’s perianal region. Thus, close physical contact may increase contamination of co-sleeping family members. Moreover, *L. edwardsi* individuals used their sleeping sites significantly longer than *A. occidentalis* (mean of 25 days vs. 4 days). A regularly re-visited, closed sleeping site presents a favorable environment for parasites with a direct life cycle, such as strongyle nematodes previously described in lemurs [50].

In contrast to *A. occidentalis*, strongyle parasites were observed in both seasons in *L. edwardsi*. This may be attributed to the favorable conditions for parasite development within tree holes, which facilitate year-round transmission of infective larvae. However, it has to be considered that despite the many ecological similarities observed between *A. occidentalis* and *L. edwardsi*, these two species belong to separate lemur families, the Indriidae and the Lepilemuridae, which have diverged 10 million years ago [65]. Coevolution of parasites with their host as well as loss of parasites over evolutionary time are important processes determining parasite assemblages [66]. Oxyurid nematodes have also been described in *L. ruficadatus* [67] and *L. dorsalis* [68], whereas to our knowledge, no oxyurid nematodes have been found in *Avahi* species. Therefore, the fact that oxyurid nematodes were only present in *L. edwardsi* might also be owed to the loss of this parasite during the evolution of *A. occidentalis*.

Even though the social behaviour of both species is similar, *L. edwardsi* is observed to engage more often in allogrooming than *A. occidentalis* [40]. Consequently, the higher parasitism in *L. edwardsi* could also be explained by its social interactions outside the sleeping holes. Thus, to verify the hypothesis that sleeping site ecology indeed affects endoparasitism, it would be necessary to analyze the effect of variation in this trait on parasite infection within the same species.

In the current study, the influence of the number of different sleeping sites (in this case tree holes) on infection status in *L. edwardsi* was tested, however, no impact of the re-use pattern of sleeping sites on infection status was observed. This is probably due to the low number of observed individuals in combination with a small variance in the number of used sleeping sites. Since it could not be determined how many individuals were sleeping together in a tree hole (except for few random nights), the influence of this factor on parasite prevalence and species diversity could not be analyzed. Further intraspecific studies on *Lepilemur* spp. or on other mammals with varying sleeping site dynamics are needed to fully understand the interaction between sleeping site ecology and parasitism. By comparing individuals of the same species one can exclude socio-behavioral and evolutionary differences.

Along with host’s behavioral properties, seasonal differences in parasite prevalence have been studied and noted repeatedly in primates [27, 69]. As expected, our study revealed a higher prevalence in the rainy season in *A. occidentalis.* This may be attributed to increased temperature and humidity during the rainy season, favoring survival and development of parasite eggs as well as larval hatching. Nevertheless, it was remarkable that strongyle prevalence in *A. occidentalis* dropped to 0% in the dry season, compared to about 38% prevalence in the rainy season. This phenomenon may be explained by an arrested development of the strongyle parasites. When conditions for external larval development are unfavorable (dry or cold), nematode larvae may undergo hypobiosis, a temporary inhibition of development at a point of their life cycle in the gut mucosa of the host [70]. This ability has been described in numerous strongyle nematodes, such as Trichostrongylidae, Ancylostomatidae and Strongylidae [71]. Arrested development is often observed in cattle and sheep and usually occurs in winter in temperate regions or in summer in hot dry regions [72]. In tropical parts of the world, like Madagascar, arrested development during the dry season may be beneficial for the parasite, as humidity is low and temperatures are cooler, hampering larval development. However, no seasonal difference in Strongylida prevalence was found in *L. edwardsi*. They were present during both seasons and do not seem to have arrested their development, possibly as a consequence of the favorable surroundings offered by tree holes as mentioned above. The oxyurid nematodes in *L. edwardsi* appeared to be unaffected by weather as well, which can be explained by the fact that the infective stages live in close proximity to the host (in the fur at the perianal region) and are therefore less dependent on the environmental circumstances like high temperature and humidity.

Finally, the effect of sex on the likelihood of infection with intestinal parasites was analyzed. No sex differences were noted in both of the study species, which is in accordance with numerous other studies [12, 73, 74]. Factors sometimes linked to higher parasite prevalence in males are the immunosuppressive effects of higher testosterone levels in males, sexual dimorphism in body size as well as sex-specific behavior [33, 34]. When behavior changes with season, i.e. males are more active during the mating season, season-specific sex differences may be observed [75]. The absence of sex differences in the presented study may not be surprising, since *A. occidentalis* and *L. edwardsi* are sexually monomorphic with regard to body size and body mass [44, 46], and there might be only small differences in testosterone levels between males and females due to female dominance [44, 76, 77].

## Conclusions

The presented study compared two primate hosts, *L. edwardsi* and *A. occidentalis,* which have a very similar lifestyle, but differ in their choice of sleeping sites (tree holes vs. branches), using them as a model to assess the influence of sleeping sites on intestinal parasite prevalence and species richness. Species diversity of intestinal parasites found in both host species was low compared to other wild primates. This may be associated, inter alia, with the arboreal lifestyle of these lemurs, limiting their contact with infective parasite stages as feces fall to the ground. *L. edwardsi* showed a higher prevalence and diversity of endoparasitic helminths than *A. occidentalis*, which might be due to the fact that this species sleeps in tree holes and shows high sleeping site fidelity, whereas *A. occidentalis* sleeps on open branches with a lower sleeping site fidelity, suggesting an influence of the sleeping site ecology on intestinal helminths. This should be verified by further intraspecific studies analyzing the influence of varying sleeping site dynamics on parasitism. Additionally, a seasonal difference in parasite infection was observed in *A. occidentalis* with strongyle egg excretion being observed solely during the rainy season. This may suggest an arrested development of strongyle nematodes harbored by *A. occidentalis* during the dry season. However, season did not seem to affect helminth infection in *L. edwardsi.* Overall, this study enhances the present knowledge of intestinal parasite communities and their determinants for nocturnal and arboreal primates.

## Additional files

**Additional file 1.** Number of sleeping site observation days per month of each radio-collared individual.

**Additional file 2.** Number of individuals sampled at different frequencies (once to six times) during the dry and the rainy season.

**Additional file 3.** Individual sampling frequency in the dry and in the rainy season.

**Additional file 4.** Number of days each individual of *L. edwardsi* spent in one sleeping site.

**Additional file 5.** Map of the forest parcel JBA showing the location of the sleeping sites of *L. edwardsi*. Every symbol represents the sleeping site of an individual or couple.

**Additional file 6.** Number of days each individual of *A. occidentalis* spent in one sleeping site.

**Additional file 7.** Map of the forest parcel JBA showing the location of the sleeping sites of *A. occidentalis*. Every symbol represents the sleeping site of a couple or family group.

**Additional file 8.** Body mass and parasite egg excretion of individuals.
