# Supplementary material for: Are sleeping site ecology and season linked to intestinal helminth prevalence and diversity in two sympatric, nocturnal and arboreal primate hosts (Lepilemur edwardsi and Avahi occidentalis)?
Source: BMC Ecol. 2018 Jul 13;18:22. doi: 10.1186/s12898-018-0178-8 (PMC6043982; doi:10.1186/s12898-018-0178-8)
Supplement: Supplementary file 1 — Additional file 1. Number of sleeping site observation days per month of each radio-collared individual. [file 12898_2018_178_MOESM1_ESM.docx]

Additional file 1: Number of sleeping site observation days per month of each radio-collared individual

| Species | Animal ID | July | August | September | October | November | March | April |
| --- | --- | --- | --- | --- | --- | --- | --- | --- |
| *L. edwardsi* | L0113 | 7 | 27 | 24 | 23 | 11 | 11 | 25 |
| *L. edwardsi* | L0213 | 7 | 27 | 24 | 23 | 11 | 11 | 25 |
| *L. edwardsi* | L0313 | 7 | 27 | 24 | 23 | 11 | 11 | 20 |
| *L. edwardsi* | L0413 | 7 | 27 | 24 | 23 | 11 | 10 | 27 |
| *L. edwardsi* | L0513 | 7 | 27 | 24 | 23 | 11 | 0 | 0 |
| *L. edwardsi* | L0713 | 5 | 27 | 24 | 23 | 11 | 11 | 25 |
| *L. edwardsi* | L0813 | 5 | 26 | 24 | 23 | 11 | 0 | 0 |
| *L. edwardsi* | L0913 | 5 | 27 | 21 | 1 | 0 | 0 | 0 |
| *L. edwardsi* | L1013 | 5 | 27 | 24 | 23 | 11 | 11 | 25 |
| *L. edwardsi* | L1231 | 5 | 27 | 24 | 23 | 11 | 10 | 22 |
| *L. edwardsi* | L1113 | 4 | 27 | 24 | 23 | 11 | 0 | 0 |
| *L. edwardsi* | L2013 | 0 | 0 | 0 | 0 | 0 | 9 | 27 |
| *L. edwardsi* | L0114 | 0 | 0 | 0 | 0 | 0 | 9 | 27 |
| *A. occidentalis* | A0113 | 6 | 26 | 23 | 23 | 11 | 0 | 0 |
| *A. occidentalis* | A0213 | 5 | 25 | 24 | 23 | 11 | 10 | 21 |
| *A. occidentalis* | A0313 | 3 | 26 | 24 | 23 | 3 | 0 | 0 |
| *A. occidentalis* | A0413 | 0 | 25 | 24 | 23 | 9 | 0 | 0 |
| *A. occidentalis* | A0513 | 0 | 24 | 23 | 17 | 7 | 0 | 0 |
| *A. occidentalis* | A0813 | 0 | 26 | 23 | 23 | 11 | 0 | 25 |
| *A. occidentalis* | A0114 | 0 | 0 | 0 | 0 | 0 | 3 | 24 |
| *A. occidentalis* | A0214 | 0 | 0 | 0 | 0 | 0 | 3 | 23 |
| *A. occidentalis* | A0314 | 0 | 0 | 0 | 0 | 0 | 2 | 22 |
