# Supplementary material for: Are sleeping site ecology and season linked to intestinal helminth prevalence and diversity in two sympatric, nocturnal and arboreal primate hosts (Lepilemur edwardsi and Avahi occidentalis)?
Source: BMC Ecol. 2018 Jul 13;18:22. doi: 10.1186/s12898-018-0178-8 (PMC6043982; doi:10.1186/s12898-018-0178-8)
Supplement: Supplementary file 2 — Additional file 2. Number of individuals sampled at different frequencies (once to six times) during the dry and the rainy season. [file 12898_2018_178_MOESM2_ESM.docx]

Additional file 2: Number of individuals sampled at different frequencies (once to six times) during the dry and the rainy season

| Sampling frequency | *A. occidentalis* | | *L. edwardsi* | |
| --- | --- | --- | --- | --- |
|  | Dry season | Rainy season | Dry season | Rainy season |
| 1x | 8 | 7 | 10 | 9 |
| 2x | 4 | 2 | 4 | 1 |
| 3x | 3 | 2 | 4 | 1 |
| 4x | 2 | 2 | 2 | 6 |
| 5x | 0 | 2 | 1 | 1 |
| 6x | 0 | 1 | 0 | 0 |
| Total | 33 | 41 | 43 | 43 |
