# Supplementary material for: Are sleeping site ecology and season linked to intestinal helminth prevalence and diversity in two sympatric, nocturnal and arboreal primate hosts (Lepilemur edwardsi and Avahi occidentalis)?
Source: BMC Ecol. 2018 Jul 13;18:22. doi: 10.1186/s12898-018-0178-8 (PMC6043982; doi:10.1186/s12898-018-0178-8)
Supplement: Supplementary file 3 — Additional file 3. Individual sampling frequency in the dry and in the rainy season. [file 12898_2018_178_MOESM3_ESM.docx]

Additional file 3: Individual sampling frequency in the dry and in the rainy season

| Species | Anima ID | Dry season | Rainy season |
| --- | --- | --- | --- |
| *L. edwardsi* | L0113 | 3 | 5 |
| *L. edwardsi* | L0213 | 2 | 4 |
| *L. edwardsi* | L0313 | 2 | 4 |
| *L. edwardsi* | L0413 | 4 | 4 |
| *L. edwardsi* | L0513 | 1 | 1 |
| *L. edwardsi* | L0713 | 4 | 4 |
| *L. edwardsi* | L0813 | 3 | 0 |
| *L. edwardsi* | L0913 | 1 | 1 |
| *L. edwardsi* | L1013 | 3 | 4 |
| *L. edwardsi* | L1113 | 3 | 1 |
| *L. edwardsi* | L1213 | 5 | 1 |
| *L. edwardsi* | L1313 | 1 | 0 |
| *L. edwardsi* | L1413 | 2 | 0 |
| *L. edwardsi* | L1513 | 2 | 1 |
| *L. edwardsi* | L1613 | 1 | 0 |
| *L. edwardsi* | L1713 | 1 | 0 |
| *L. edwardsi* | L1813 | 1 | 0 |
| *L. edwardsi* | L1913 | 1 | 2 |
| *L. edwardsi* | L2013 | 1 | 3 |
| *L. edwardsi* | L2113 | 1 | 0 |
| *L. edwardsi* | L2213 | 1 | 1 |
| *L. edwardsi* | L2313 | 1 | 0 |
| *L. edwardsi* | L0114 | 0 | 4 |
| *L. edwardsi* | L0214 | 0 | 1 |
| *L. edwardsi* | L0314 | 0 | 1 |
| *L. edwardsi* | L0414 | 0 | 1 |
| *L. edwardsi* | Lp0413 | 1 | 0 |
| *L. edwardsi* | L0514 | 0 | 1 |
| *A. occidentalis* | A0113 | 3 | 1 |
| *A. occidentalis* | A0213 | 3 | 4 |
| *A. occidentalis* | A0313 | 4 | 0 |
| *A. occidentalis* | A0413 | 4 | 2 |
| *A. occidentalis* | A0513 | 3 | 0 |
| *A. occidentalis* | A0613 | 2 | 0 |
| *A. occidentalis* | A0713 | 1 | 0 |
| *A. occidentalis* | A0813 | 2 | 1 |
| *A. occidentalis* | A0913 | 2 | 1 |
| *A. occidentalis* | A1013 | 1 | 0 |
| *A. occidentalis* | A1113 | 0 | 6 |
| *A. occidentalis* | A1213 | 1 | 0 |
| *A. occidentalis* | A1313 | 2 | 3 |
| *A. occidentalis* | A1413 | 1 | 0 |
| *A. occidentalis* | A1613 | 1 | 0 |
| *A. occidentalis* | A1713 | 1 | 0 |
| *A. occidentalis* | A1813 | 1 | 0 |
| *A. occidentalis* | Af0513 | 1 | 0 |
| *A. occidentalis* | As0113 | 0 | 1 |
| *A. occidentalis* | A1913 | 1 | 0 |
| *A. occidentalis* | A0114 | 0 | 4 |
| *A. occidentalis* | A0214 | 0 | 5 |
| *A. occidentalis* | A0314 | 0 | 5 |
| *A. occidentalis* | Af0314 | 0 | 1 |
| *A. occidentalis* | A0414 | 0 | 3 |
| *A. occidentalis* | A0514 | 0 | 1 |
| *A. occidentalis* | A0614 | 0 | 1 |
| *A. occidentalis* | A0714 | 0 | 2 |
| *A. occidentalis* | A0814 | 0 | 1 |
