# Supplementary material for: Are sleeping site ecology and season linked to intestinal helminth prevalence and diversity in two sympatric, nocturnal and arboreal primate hosts (Lepilemur edwardsi and Avahi occidentalis)?
Source: BMC Ecol. 2018 Jul 13;18:22. doi: 10.1186/s12898-018-0178-8 (PMC6043982; doi:10.1186/s12898-018-0178-8)
Supplement: Supplementary file 4 — Additional file 4. Number of days each individual of L. edwardsi spent in one sleeping site. [file 12898_2018_178_MOESM4_ESM.docx]

Additional file 4: Number of days each individual of *L. edwardsi* spent in one sleeping site

| Animal ID | L0113 | L0213 | L0313 | L0413 | L0513 | L0713 | L0813 | L0913 | L1013 | L1213 | L1113 | L2013 | L0114 |
| --- | --- | --- | --- | --- | --- | --- | --- | --- | --- | --- | --- | --- | --- |
| Site 1 | 107 | 107 | 83 | 6 | 3 | 4 | 23 | 54 | 30 | 3 | 1 | 15 | 3 |
| Site 2 | 1 | 15 | 1 | 26 | 22 | 5 | 57 |  | 92 | 106 | 1 | 21 | 32 |
| Site 3 | 4 | 1 | 1 | 8 | 66 | 1 | 7 |  | 4 | 6 | 85 |  | 1 |
| Site 4 | 12 | 4 | 35 | 44 |  | 104 | 9 |  |  | 5 | 2 |  |  |
| Site 5 | 1 | 1 | 3 | 45 |  | 12 |  |  |  | 2 |  |  |  |
| Site 6 | 3 |  |  |  |  | 1 |  |  |  |  |  |  |  |
