# Supplementary material for: Are sleeping site ecology and season linked to intestinal helminth prevalence and diversity in two sympatric, nocturnal and arboreal primate hosts (Lepilemur edwardsi and Avahi occidentalis)?
Source: BMC Ecol. 2018 Jul 13;18:22. doi: 10.1186/s12898-018-0178-8 (PMC6043982; doi:10.1186/s12898-018-0178-8)
Supplement: Supplementary file 5 — Additional file 5. Map of the forest parcel JBA showing the location of the sleeping sites of L. edwardsi. Every symbol represents the sleeping site of an individual or couple. [file 12898_2018_178_MOESM5_ESM.docx]

Additional file 5: Map of the forest parcel JBA showing the location of the sleeping sites of *L. edwardsi*. Every symbol represents the sleeping site of an individual or couple.
