# Supplementary material for: Are sleeping site ecology and season linked to intestinal helminth prevalence and diversity in two sympatric, nocturnal and arboreal primate hosts (Lepilemur edwardsi and Avahi occidentalis)?
Source: BMC Ecol. 2018 Jul 13;18:22. doi: 10.1186/s12898-018-0178-8 (PMC6043982; doi:10.1186/s12898-018-0178-8)
Supplement: Supplementary file 6 — Additional file 6. Number of days each individual of A. occidentalis spent in one sleeping site. [file 12898_2018_178_MOESM6_ESM.docx]

Additional file 6: Number of days each individual of *A. occidentalis* spent in one sleeping site

| Animal ID | A0113 | A0213 | A0313 | A0413 | A0513 | A0813 | A0114 | A0214 | A0314 |
| --- | --- | --- | --- | --- | --- | --- | --- | --- | --- |
| Site 1 | 19 | 2 | 20 | 4 | 26 | 11 | 11 | 2 | 2 |
| Site 2 | 2 | 1 | 3 | 16 | 19 | 1 | 1 | 1 | 2 |
| Site 3 | 1 | 1 | 1 | 1 | 1 | 1 | 1 | 2 | 7 |
| Site 4 | 19 | 5 | 1 | 1 | 2 | 3 | 1 | 4 | 1 |
| Site 5 | 1 | 11 | 1 | 1 | 1 | 3 | 1 | 1 | 11 |
| Site 6 | 7 | 1 | 1 | 1 | 2 | 5 | 1 | 11 | 1 |
| Site 7 | 2 | 4 | 1 | 9 | 3 | 3 | 3 | 2 |  |
| Site 8 | 9 | 1 | 4 | 10 | 6 | 14 | 1 | 2 |  |
| Site 9 | 1 | 1 | 28 | 4 | 2 | 3 | 3 | 1 |  |
| Site 10 | 24 | 1 | 2 | 1 | 1 | 3 | 1 |  |  |
| Site 11 | 3 | 18 | 1 | 2 | 4 | 1 | 2 |  |  |
| Site 12 | 1 | 1 | 1 | 1 | 4 | 10 | 1 |  |  |
| Site 13 |  | 1 | 6 | 9 |  | 1 |  |  |  |
| Site 14 |  | 3 | 7 | 8 |  | 1 |  |  |  |
| Site 15 |  | 3 | 1 | 4 |  | 1 |  |  |  |
| Site 16 |  | 10 |  | 3 |  | 1 |  |  |  |
| Site 17 |  | 4 |  | 2 |  | 1 |  |  |  |
| Site 18 |  | 1 |  | 2 |  | 1 |  |  |  |
| Site 19 |  | 1 |  | 1 |  | 2 |  |  |  |
| Site 20 |  | 12 |  |  |  | 1 |  |  |  |
| Site 21 |  | 1 |  |  |  | 4 |  |  |  |
| Site 22 |  | 7 |  |  |  | 5 |  |  |  |
| Site 23 |  | 1 |  |  |  | 1 |  |  |  |
| Site 24 |  | 2 |  |  |  | 2 |  |  |  |
| Site 25 |  | 23 |  |  |  | 1 |  |  |  |
| Site 26 |  | 2 |  |  |  | 3 |  |  |  |
| Site 27 |  |  |  |  |  | 2 |  |  |  |
| Site 28 |  |  |  |  |  | 2 |  |  |  |
| Site 29 |  |  |  |  |  | 10 |  |  |  |
