# Supplementary material for: Are sleeping site ecology and season linked to intestinal helminth prevalence and diversity in two sympatric, nocturnal and arboreal primate hosts (Lepilemur edwardsi and Avahi occidentalis)?
Source: BMC Ecol. 2018 Jul 13;18:22. doi: 10.1186/s12898-018-0178-8 (PMC6043982; doi:10.1186/s12898-018-0178-8)
Supplement: Supplementary file 8 — Additional file 8. Body mass and parasite egg excretion of individuals. [file 12898_2018_178_MOESM8_ESM.docx]

Additional file 8: Body mass and parasite egg excretion of individuals

| Species | Season | Anima ID | Body mass in g | Animal sex | Parasite egg excretion |
| --- | --- | --- | --- | --- | --- |
| *L. edwardsi* | Dry | L0113 | 940 | F | No |
| *L. edwardsi* | Dry | L0213 | 868 | M | Yes |
| *L. edwardsi* | Dry | L0313 | 780 | M | No |
| *L. edwardsi* | Dry | L0413 | 624 | F | Yes |
| *L. edwardsi* | Dry | L0513 | 1050 | F | Yes |
| *L. edwardsi* | Dry | L0713 | 954 | M | Yes |
| *L. edwardsi* | Dry | L0813 | 1010 | M | Yes |
| *L. edwardsi* | Dry | L1013 | 876 | F | Yes |
| *L. edwardsi* | Dry | L1113 | 564 | F | Yes |
| *L. edwardsi* | Dry | L1213 | 888 | M | Yes |
| *L. edwardsi* | Dry | L1313 | 946 | F | No |
| *L. edwardsi* | Dry | L1413 | 884 | M | No |
| *L. edwardsi* | Dry | L1513 | 1050 | F | No |
| *L. edwardsi* | Dry | L1613 | 843 | M | Yes |
| *L. edwardsi* | Dry | L1713 | 856 | F | No |
| *L. edwardsi* | Dry | L1813 | 838 | M | No |
| *L. edwardsi* | Dry | L1913 | 710 | M | Yes |
| *L. edwardsi* | Dry | L2013 | 926 | M | No |
| *L. edwardsi* | Dry | L2213 | 852 | M | Yes |
| *L. edwardsi* | Rainy | L0113 | 858 | F | Yes |
| *L. edwardsi* | Rainy | L0114 | 944 | F | Yes |
| *L. edwardsi* | Rainy | L0213 | 868 | M | Yes |
| *L. edwardsi* | Rainy | L0214 | 943 | F | Yes |
| *L. edwardsi* | Rainy | L0313 | 836 | M | Yes |
| *L. edwardsi* | Rainy | L0414 | 994 | F | Yes |
| *L. edwardsi* | Rainy | L0513 | 1050 | F | No |
| *L. edwardsi* | Rainy | L0713 | 1018 | M | Yes |
| *L. edwardsi* | Rainy | L0913 | 864 | F | No |
| *L. edwardsi* | Rainy | L1013 | 1078 | F | Yes |
| *L. edwardsi* | Rainy | L1113 | 564 | F | No |
| *L. edwardsi* | Rainy | L1213 | 902 | M | No |
| *L. edwardsi* | Rainy | L1513 | 976 | F | No |
| *L. edwardsi* | Rainy | L1913 | 964 | M | Yes |
| *L. edwardsi* | Rainy | L2013 | 808 | M | Yes |
| *L. edwardsi* | Rainy | L2213 | 944 | M | No |
| *A. occidentalis* | Dry | A0113 | 996 | F | No |
| *A. occidentalis* | Dry | A0213 | 1104 | F | No |
| *A. occidentalis* | Dry | A0313 | 806 | M | No |
| *A. occidentalis* | Dry | A0413 | 836 | M | No |
| *A. occidentalis* | Dry | A0613 | 882 | F | No |
| *A. occidentalis* | Dry | A0713 | 840 | F | No |
| *A. occidentalis* | Dry | A0813 | 1002 | F | No |
| *A. occidentalis* | Dry | A0913 | 770 | M | No |
| *A. occidentalis* | Dry | A1213 | 794 | M | No |
| *A. occidentalis* | Dry | A1313 | 770 | M | No |
| *A. occidentalis* | Dry | A1813 | 798 | M | No |
| *A. occidentalis* | Rainy | A0113 | 996 | F | No |
| *A. occidentalis* | Rainy | A0114 | 846 | F | Yes |
| *A. occidentalis* | Rainy | A0213 | 1120 | F | Yes |
| *A. occidentalis* | Rainy | A0214 | 939 | F | Yes |
| *A. occidentalis* | Rainy | A0314 | 982 | M | Yes |
| *A. occidentalis* | Rainy | A0413 | 836 | M | No |
| *A. occidentalis* | Rainy | A0514 | 898 | F | No |
| *A. occidentalis* | Rainy | A0614 | 770 | F | No |
| *A. occidentalis* | Rainy | A0714 | 726 | M | No |
| *A. occidentalis* | Rainy | A0813 | 1002 | F | No |
| *A. occidentalis* | Rainy | A0913 | 770 | M | No |
| *A. occidentalis* | Rainy | A1113 | 906 | M | Yes |
| *A. occidentalis* | Rainy | A1313 | 770 | M | No |
